# *Nigella sativa* L. seed extracts promote wound healing progress by activating VEGF and PDGF signaling pathways: An *in vitro* and *in silico* study

**DOI:** 10.12688/f1000research.132106.2

**Published:** 2023-06-21

**Authors:** Chella Perumal Palanisamy, Phaniendra Alugoju, Selvaraj Jayaraman, Sirilux Poompradub

**Affiliations:** 1 Department of Chemical Technology, Faculty of Science, Chulalongkorn University, Bangkok, 10330, Thailand; 2 Natural Products for Neuroprotection and Anti-Ageing Research Unit, Chulalongkorn University, Bangkok, 10330, Thailand; 3 Centre of Molecular Medicine and Diagnostics (COMManD), Department of Biochemistry, Saveetha Dental College & Hospital, Saveetha Institute of Medical & Technical Sciences, Saveetha University, Chennai, 600077, India; 4 Center of Excellence in Green Materials for Industrial Application, Faculty of Science, Chulalongkorn University, Bangkok, Thailand

**Keywords:** Nigella sativa L., Wound healing, Vascular endothelial growth factor, Platelet-derived growth factor

## Abstract

**Background:** A significant area of clinical research is the development of natural wound healing products and the management of chronic wounds. Healing wounds with medicinal plants has been a practice of ancient civilizations for centuries. *Nigella sativa* L ( *N. sativa*) is a medicinal plant that has several pharmacological properties.

**Methods:** The present study evaluated the wound healing properties of *Nigella sativa* L. ( *N. sativa*) seed extracts using normal cell lines such as normal human dermal fibroblasts (NHDFs) and human umbilical vein endothelial cells (HUVECs). The expression levels of vascular endothelial growth factor (VEGF) and platelet-derived growth factor (PDGF) were analyzed through western blot analysis. Furthermore, computational analyses were carried out to screen the potential bioactive compounds for wound healing applications.

**Results:** The results of the 3-(4,5-dimethylthiazol-2-yl)-5-(3-carboxymethoxyphenyl)-2-(4-sulfophenyl)-2H-tetrazolium (MTS) assay revealed that, all the tested solvent extracts of *N. sativa* seeds (including ethanol, ethyl acetate, chloroform, and petroleum ether) did not exert any cytotoxic effects at the tested concentrations. Furthermore, the western blot analysis showed elevated levels of VEGF and PDGF upon treatment with *N. sativa* seed extracts. Gas chromatography-mass spectrometry (GC-MS) analysis of *N. sativa *extracts identified 268 phytocompounds. Molecular docking studies revealed that three phytocompounds of *N. sativa* extracts, including tricyclo[20.8.0.0(7,16)]triacontane, 1(22),7(16)-diepoxy-, adaphostin and obeticholic acid had strong binding affinity with wound healing-related target proteins, showing docking scores ranging from -5.5 to -10.9 Kcal/mol. These compounds had acceptable Absorption, Distribution, Metabolism, and Excretion (ADME) properties.

**Conclusions:** Based on these results, *N. sativa* seed extracts might possess potential wound healing properties owing to the presence of a wide range of bioactive components.

## Introduction

The management of wounds, especially extensive and full-thickness wounds, has long been a concern in the field of medicine. ^1^ It is possible that infection by pathogenic bacteria delays wound healing and poses a health risk to the general public. Clinicians exploring effective ways to promote wound healing is a hot topic in research. ^2^ Vigorous development of advanced wound dressings is imperative for accelerating wound healing and achieving closure of wounds quickly. ^3^ Hemostasis, inflammation, proliferation, and tissue remodeling are sequential and timed processes involved in wound healing. ^4^ These complex processes are mediated by released cytokines, chemokines, and growth factors, which are released by neutrophils, macrophages, keratinocytes, and endothelial cells. ^5^ It is important to manage wounds in a timely and comfortable manner in order to facilitate a quick healing process. ^6^ The wound care industry has developed a number of products that are designed to treat wounds (for example MEBO, Calmoseptine ^®^, Boroline). A variety of wound healing techniques have been developed over the years, including traditional (especially herbal) and modern methods. ^1^^,^^7^ Traditional herbal wound-healing therapies remain popular among rural populations in developing countries in part due to their availability and affordability, and they have been demonstrated to be effective, clinically accepted, and have few or no side effects. ^8^

There has been a growing awareness in recent years that many phytocompounds possess medicinal properties that are effective in treating diseases and in healing wounds. ^9^ A chemical scaffold can provide a framework for developing synthetic and/or semi-synthetic analogues of drugs, which can be used in drug development for disease treatment in a wide range of settings. ^10^ As a result of the advent of modern techniques like molecular biology, metabolomics, phytochemical analysis, and drug discovery, natural products chemists have been able to unravel the ancient therapeutic hypotheses and mechanisms of herbal medicines. ^11^^–^^14^ It is common to find these types of treatments used in Ayurveda, Traditional Chinese medicine, and Traditional Thai medicine. ^15^

*Nigella sativa* L. ( *N. sativa*) (Family Ranunculaceae) seeds, commonly known as black cumin or black seeds, have a long history of being used as a treatment for a variety of aliments by traditional healers throughout the world, in regions like South-eastern Asia, the Middle East, Africa, and many areas of the Mediterranean. ^16^^,^^17^ It is also notable to point out that *N. sativa* possess a plethora of pharmacological properties. A variety of health-related conditions have been treated with this herb throughout history, including respiratory and digestive disorders, and kidney, liver, and cardiovascular diseases. ^18^ The most important pharmacological effects of *N. sativa* seeds can be attributed to thymoquinone, according to a previous study. ^19^ The extracts of *N. sativa* seeds also contain alkaloids, saponins, steroids, terpenoids, p-cymene, limonene, and fatty acids as well as proteins, carbohydrates, vitamins, trace minerals (like iron and zinc) and crude fiber. ^20^

*N. sativa* seeds are reported to have several pharmacological effects, including analgesic, appetizer, anti-diabetic, antioxidant, anti-inflammatory, and antimicrobial properties. ^21^ Despite extensive research on the phytochemical pharmacological properties of *N. sativa* seeds, *N. sativa* seed extracts are not yet completely characterized chemically. ^22^ As people become more aware that natural products can have potential therapeutic effects on wound healing properties and at present do not have any known toxic effects, it is becoming clear that they are seeking out natural products that can work in this regard. As well as providing information regarding the compositional profile and evaluating the medicinal effects of herbal extracts and/or oils, there is a need to re-evaluate their therapeutic properties in addition to providing information regarding their compositional profile. ^23^ Consequently, the primary objective of the present study was to investigate the effects of *N. sativa* seed extracts and their phytocompounds on wound healing. Normal human dermal fibroblasts (NHDFs) and human umbilical vein endothelial cells (HUVECs) were used as primary cell lines in the present study to study these issues *in vitro.* The phytochemicals were also docked with multiple wound healing-related proteins (Tumor necrosis factor α (TNFα), transforming growth factor beta receptor 1 (TGFBR1) kinase, interleukin-1 beta (IL-1β), protein kinase C (PKC)-βII, vascular endothelial growth factor (VEGF) and platelet-derived growth factor (PDGF)).

## Methods

This study was performed at Chulalongkorn University (Thailand) and Saveetha University (India).

### Chemicals

Bovine serum albumin (BSA) (cat. no. 23209), HUVECs (cat. no. C0035C) and NHDFs (cat. no. C0135C) used in this study were purchased from Thermo Fisher Scientific Inc. Dulbecco’s Modified Eagle Medium (DMEM) (cat. no. D6429), TRIS-buffered saline (TBS) (cat. no. SRE0071) and fetal bovine serum (FBS) (cat. no. F7524) were purchased from Sigma-Aldrich. 3-(4,5-dimethylthiazol-2-yl)-5-(3-carboxymethoxyphenyl)-2-(4-sulfophenyl)-2H-tetrazolium (MTS) was purchased from BioVision Inc. (cat. no. 2808). Anti-VEGF mouse monoclonal antibody was procured from Santa Cruz Biotechnology, Inc., (cat. no. sc-53462). Anti-PDGF Receptor β polyclonal antibody (produced in rabbit) was purchased from Sigma-Aldrich (cat. no. SAB4502149). Anti-β-actin mouse monoclonal antibody was purchased from Santa Cruz Biotechnology, Inc. (cat. no. sc-69879). Enhanced Chemiluminescence Detection (ECL) kit was obtained from Amersham BioSciences UK Ltd (cat. no. RPN2209).

### Collection of materials

*N. sativa* seeds were purchased from a local herbal shop in Bangkok, Thailand. After being cleaned with tap water, they were dried under shade conditions, powdered, and air-tight packaged in a container.

### Extract preparation

In order to determine the yield on the plant material, 3,000 mL petroleum ether, chloroform, ethyl acetate, and ethanol were continuously shocked with 600 g plant material in a conical flask for 72 h (during the cold percolation process). After the extracts were collected and filtered using Whatman No. 1 filter paper, a rotary evaporator set at 40°C was used to dry them. In order to preserve the dried extracts until further use, they were stored at 4°C until use.

### GC-MS analysis and phytocompounds identification

Gas Chromatography-Mass Spectrometer Model Shimadzu GCMS-QP2020 NX (Shimadzu, Japan) equipped with 5 Sil MS 5% diphenyl/95% dimethyl polysiloxane capillary column (measuring 30 mm wide, 0.25 mm diameter, and 0.25 mm thick) was used analyze the extracts of *N. sativa* seeds. Then, 100 μl of solvent extracts were diluted using 1,400 μl dimethyl sulfoxide (DMSO). Next, 1 μl diluted sample (100/1,400, V/V in DMSO) was injected in the split mode with a split ratio 1:10. Electron impact ionization was used for GC-MS detection with an ionization energy of 70 eV. A low flow rate of 1.0 mL per min of helium at a low pressure was used as the carrier gas in the column. Before the injector temperature was set at 250°C, 60°C was set for 15 min before gradually increasing to 280°C over 3 min. It was conducted at 70 eV with a scanning distance of 0.5 s as well as fragment sizes ranging between 50 Da and 650 Da for the MS analysis, 40 min were spent on the GC operation. Acquisition mode scan ranged from 35 m/z to 500 m/z with scan speed 2,500. Extracts were analyzed for their percentage composition of compounds. NIST20R and Wiley libraries were used to interpret and compare GC-MS data as well as compare retention indices. ^24^

### *In vitro* cell line study

*Cell lines and culture*

HUVEC and NHDF cell lines were maintained at 37°C during the experiment using a humidified atmosphere containing 5% CO _2_. DMEM supplemented with 10% FBS and 1% antibiotics (100 U/mL penicillin and 100 g/mL streptomycin) was used to grow the cells in T-25 flasks. Trypsinization and passage were performed once the cells reached 70% confluency.

*Cell viability analysis*

In culture media, 10 mg/mL stock solutions of plant extracts were diluted in DMSO. To determine cell viability, cells were seeded into 96 well plates at a density of 5x10 ^3^ cells per well and incubated at 37°C and 5% CO _2_ for 24 h. Fresh DMEM supplemented with various solvent extracts of *N. sativa* seeds (petroleum ether, chloroform, ethyl acetate and ethanol) (0, 10, 20, 50, 100 μg/mL) was added, and incubation was carried out for 24 h. After the incubation with extracts, cells were incubated for 2 h in growth media (DMEM) containing 20% MTS solution to assess viability. Microplate readers were used to measure the absorbance of formazan at 490 nm. The crude extracts were dissolved in 0.5% DMSO, which represents the highest concentration of DMSO used in the vehicle culture medium.

*Protein expression analysis by western blotting*

Laemmli (1970) described sodium dodecyl sulfate-polyacrylamide gel electrophoresis (SDS-PAGE) as a method for separating proteins. ^25^ Using equal volumes (50 g) of samples and buffer, sample mixtures were heated at 95°C for 4 min, then cooled on ice. The dye front was reached at the bottom of the running gel after separating proteins with a Bio-Rad mini slab gel apparatus at a constant voltage of 100 V. In this experiment, polyvinylidene difluoride (PVDF) membranes were charged at a constant voltage of 100 V for 1 h in order to transfer protein bands. Incubation with primary antibodies (anti-VEGF (mouse monoclonal antibody 200 μg/mL) and anti-PDGF Receptor β (rabbit polyclonal antibody 100 μg/mL); β-actin (mouse monoclonal antibody 100 μg/mL) at appropriate dilutions followed by blocking with 5% BSA blocking solution at room temperature for 1 h. A secondary antibody (goat anti-mouse monoclonal antibody 400 μg/mL), purchased from Santa Cruz Biotechnology, Inc., (cat. no. sc-2005; 1:10,000) was incubated for 1 h after primary antibody incubation. Incubation with secondary antibody was followed by two washes (5 min each) with Tris-buffered saline, Tween (TBS-T) and placement on Saran Wrap™ (protein-side up). After adding detection reagent mixture to the blot, blots were incubated for 30-60 sec and we drained off excess reagent (ECL). Quantity One 1-D Analysis Software (RRID:SCR_014280) (Bio-Rad) was used to quantify the immunoblot signals. Using a probe consisting of β-actin, similar amounts of proteins were loaded onto the membranes.

### *In silico* analysis

*Selection and preparation of ligands*

Through GC-MS analysis, a total of 268 phytocompounds were identified in four different extracts of *N. sativa.* The 3D structures of all the identified compounds were extracted from the PubChem database (RRID:SCR_004284). ^26^ A list of phytocompounds identified are provided in Tables 1-4 as *Underlying data*. ^42^ Using PyRx software (RRID:SCR_018548) with default parameters, energy minimization of each ligand was performed using universal force fields, followed by Gasteiger charges to achieve a good structural conformation for docking.

*Selection and preparation of receptors*

As part of this study, six different proteins such as TNFα, TGFBR1 kinase, IL-1β, PKC-βII, VEGF and PDGF that participate in wound healing were selected, and their crystal structures were retrieved from Protein Data Bank (PDB). Using Chimera 1.16 (RRID:SCR_002959), any missing residues in the selected target proteins were modelled, nonstandard hetero atoms were removed, polar hydrogens and Gasteiger charges were added, and then energy minimization of each protein performed with 100 steepest descent gradient steps using amber force field (Amber ff14SB). Finally, the energy minimized protein was converted into pdbqt format for molecular docking.

*Protein-ligand docking*

The Autodock Vina (RRID:SCR_011958) was used for the molecular docking of phytocompounds of *N. sativa* with selected wound healing target proteins. If the ligand binding site is represented, it will be located at the center of the grid box. A value of eight is set for the exhaustiveness of the model. A configuration file was created based on the dimensions of the XYZ axis determined by Discovery studio’s visualizer. In Autodock Vina 1.1.2, this configuration file was used for docking using the command line. To dock ligands with a degree of flexibility, Autodock Vina uses the Monte Carlo algorithm. Monte Carlo algorithm used in Autodock Vina is relatively faster than other docking programs. ^27^ In addition to the results file, the binding modes were generated as a single file (PDBQT format) in a log format. BIOVIA Discovery Studio (RRID:SCR_015651) visualizer was used to analyze the binding interactions between best docked ligands and receptors. Strong hydrogen bonds (2.2 to 2.5), moderate hydrogen bonds (2.5 to 3.2), and weak hydrogen bonds (up to 3.6) were measured with respect to the hydrogen atom of the heavy atom.

*ADME properties prediction*

QikProp (RRID:SCR_014906) module was used to predict ADME properties (Schrodinger Suite 2022). To determine a ligand’s pharmacokinetics and pharmacodynamics, the QikProp module analyses its properties, which are resembling those of a drug. Several ADME properties were considered significant, including the molecular weight (MW), H-bond donor, H-bond acceptor, and logarithm of n-octanol/water partition coefficient (log P (O/W)).

### Statistical analysis

Data were analyzed using GraphPad Prism (RRID:SCR_002798) version 5 software to assess the significance of individual variations between the control and treatment groups by one-way analysis of variance (ANOVA) and Duncan’s multiple range test. Approximately P<0.05 was considered significant in Duncan’s test.

## Results

### GC-MS analysis

**Figure 1.**
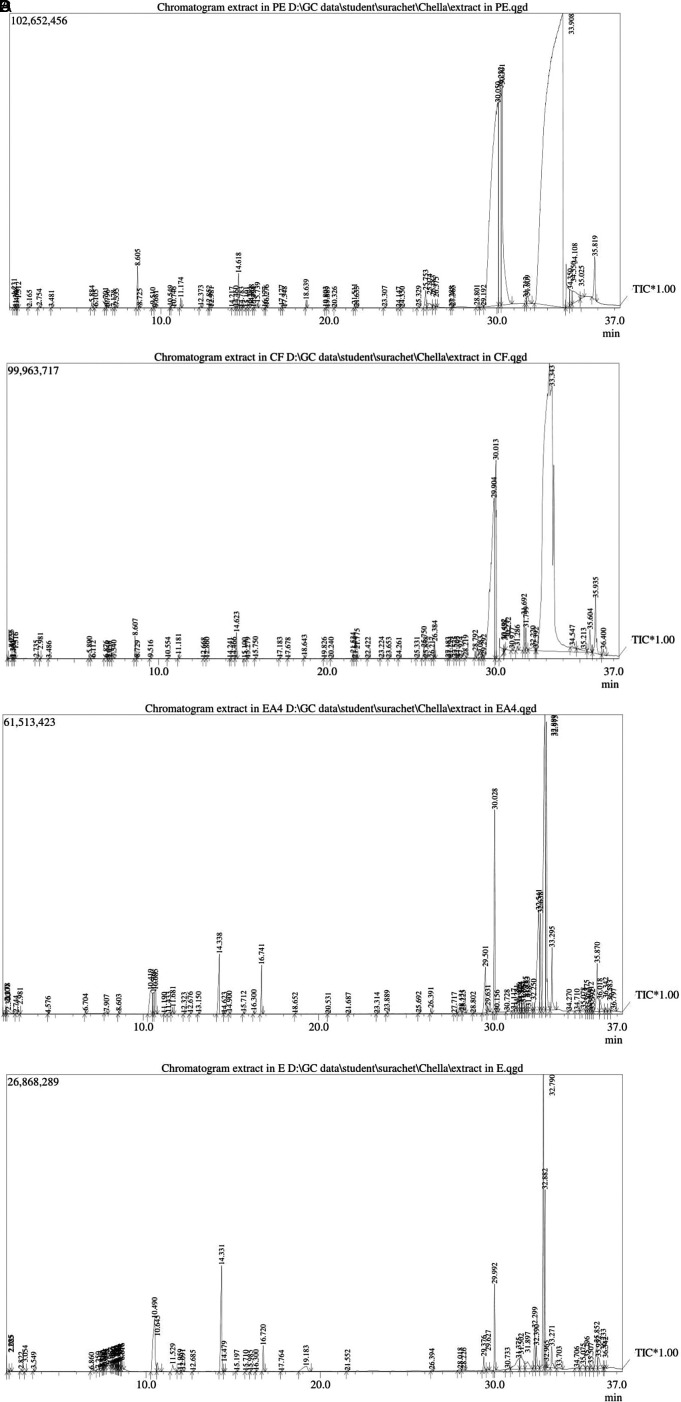
Analysis of different solvent extracts of *N. sativa* seeds using GC-MS.

GC-MS analysis identified a total of 268 phytocompounds in *N. sativa* seed extracts ( Figure 1). ^42^ Petroleum ether, chloroform, ethyl acetate and ethanolic extracts showed 65, 70, 67 and 66 peaks, respectively, which are indicating the presence of phytocompounds (Tables 1-4 in *Underlying data* ^42^). Among these, the highest peak levels were observed such as, 66.81% linoleic acid (PubChem CID: 5280450) at 33.908 min retention time (petroleum ether extract), 42% cis-vaccenic acid (PubChem CID: 5282761) at 33.343 min retention time (chloroform extract), 29.24% ethyl palmitate (PubChem CID: 12366) at 30.050 min retention time (petroleum ether extract), 20.09% oleic acid (PubChem CID: 445639) at 34.550 min retention time (petroleum ether extract), 16.72% palmitic acid (PubChem CID: 985) at 29.904 min retention time (chloroform extract), 16.57% tetradecanoic acid (PubChem CID: 11005) at 25.974 min retention time (petroleum ether extract), 16.37% 3-(3-Chlorophenyl)imidazolidine-2,4-dione (PubChem CID: 285803) at 31.375 min retention time (ethanolic extract), 15.83% methyl linoleate (PubChem CID: 5284421) at 32.22 min retention time (chloroform extract), 15.34% adaphostin (PubChem CID: 387042) at 30.733 min retention time (ethanolic extract), 14.07% glyceryl diacetate 2-oleate (PubChem CID: 5363238) at 31.897 min retention time (ethanolic extract), 12.62% 2-linoleoylglycerol (PubChem CID: 5365676) at 31.502 min retention time (ethanolic extract), 11.34% monopalmitin (PubChem CID: 14900) at 28.131 min retention time (ethyl acetate extract), 11.14% (z)-tetradec-7-enal (PubChem CID: 5364468) at 2.3 min retention time (ethyl acetate extract), 10.54% glycerol, 2-octadecanoate, diacetate (PubChem CID: 539925) at 33.703 min retention time (ethanolic extract), 10.52% glyceryl diacetate 1-linolenate (PubChem CID: 6434505) at 35.075 min retention time (ethanolic extract), 10.45% olealdehyde (PubChem CID: 5364492) at 36.797 min retention time (ethyl acetate extract), 10.25% 2,3-dihydroxypropyl acetate (PubChem CID: 33510) at 10.419 min retention time (ethyl acetate extract), 10.23% 16-trimethylsilyloxy-9-octadecenoic acid, methyl ester (PubChem CID: 6421149) at 34.547 min retention time (chloroform extract), 10.08% propyl ester (PubChem CID: 221069) at 34.71 min retention time (ethyl acetate extract).

### Effect of *N. sativa* seeds on cell viability of NHDFs and HUVECs

**Figure 2.**
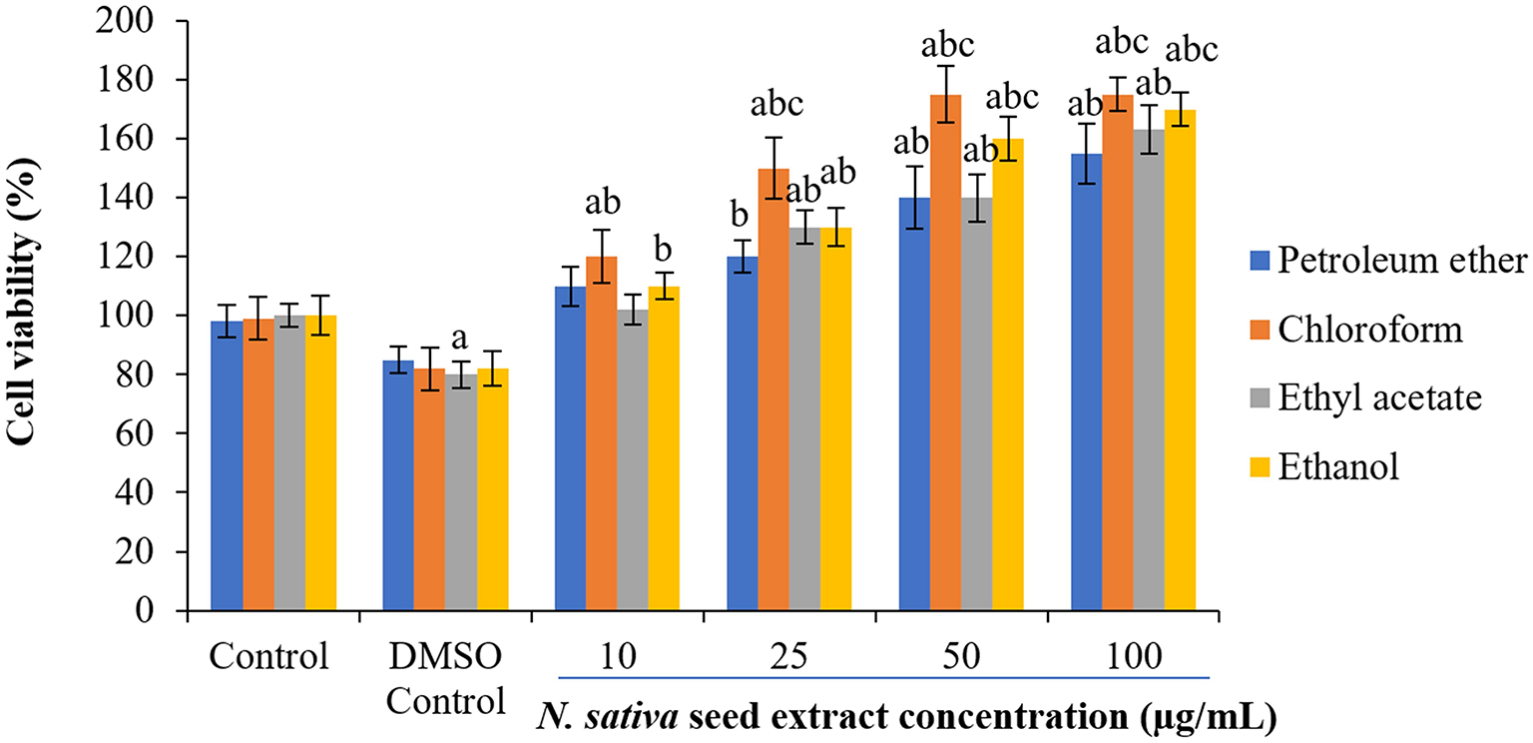
Effect of *N. sativa* seed extracts on cell viability in HUVEC cells. Cells were cultured in DMEM supplemented with 10% FBS were incubated with indicated concentrations of extracts (0–100 μg) for 24 h. Each bar represents the mean ± SEM of six independent observations. Significance was considered as the levels of p < 0.05 level using Duncan’s multiple range test. a - compared to control; b - compared to DMSO control; c - compared with 10 μg treated cells; d - compared with 25 μg treated cells.

**Figure 3.**
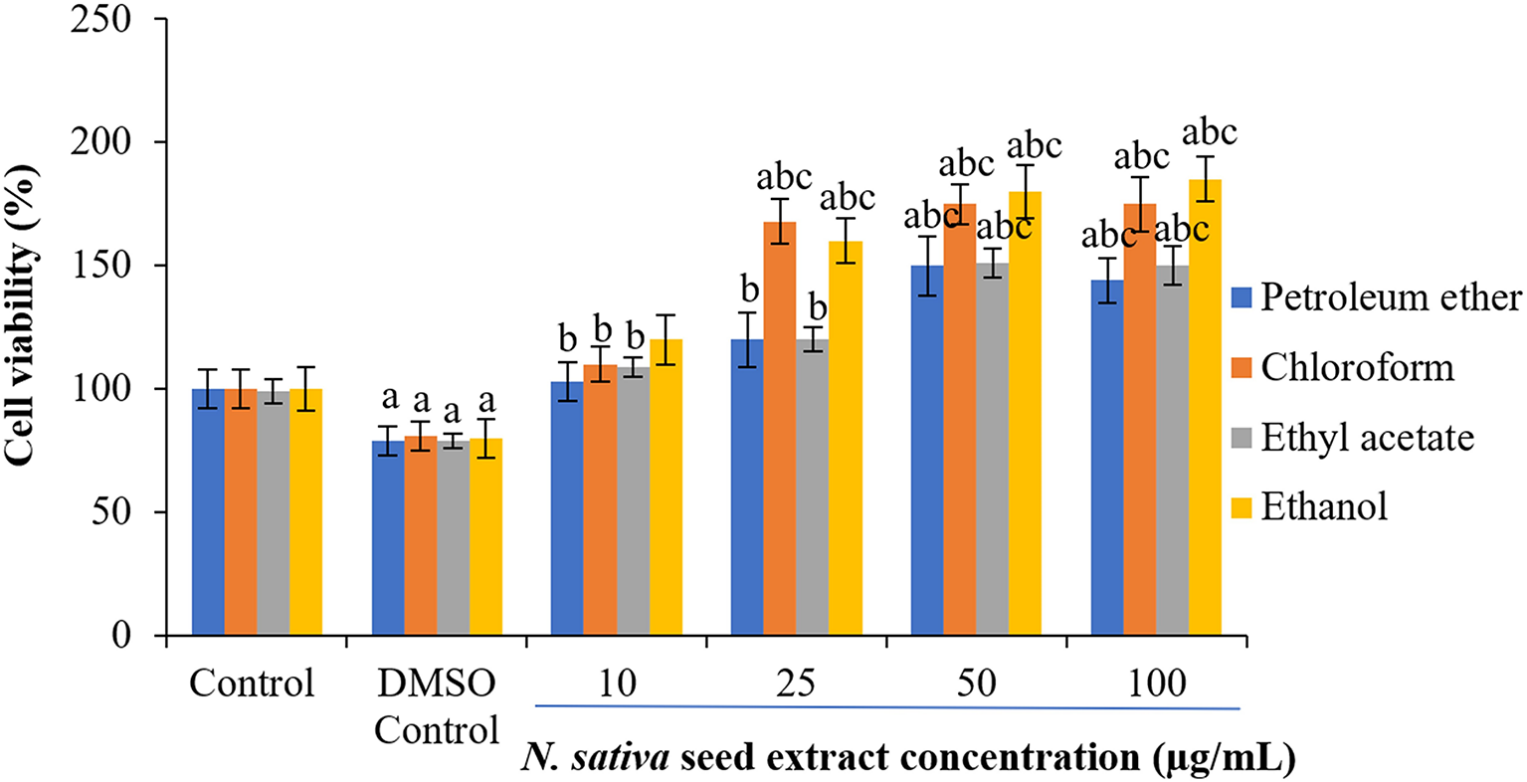
Effect of *N. sativa* seed extracts on cell viability in NHDF cells. Cells cultured in DMEM supplemented with 10% FBS were incubated with indicated concentrations of extracts (0–100 μg) of 24 h. Each bar represents the mean ± SEM of six independent observations. Significance was considered at p < 0.05 level using Duncan’s multiple range test. a - compared to control; b - compared to DMSO control; c - compared with 10 μg treated cells; d - compared with 25 μg treated cells.

To check the cytotoxicity of *N. sativa* seed extracts, two different normal cell lines (NHDF and HUVECs) were used at the different concentrations of crude extracts. Cell viability percentages were plotted against extracts treatment concentrations to obtain treatment-response curves. A concentration-dependent increase in cell viability was observed with *N. sativa* seed extracts ( Figures 2 and 3). In response to 25-50 μg/mL of ethanolic and chloroform extract on both cell lines, the viability of cells were increased by 60-68% following 24 h treatment ( Figures 2 and 3).

### Protein expression analysis

*Effect of N. sativa seed extracts on VEGF and PDGF protein expression in NHDF cells*

**Figure 4.**
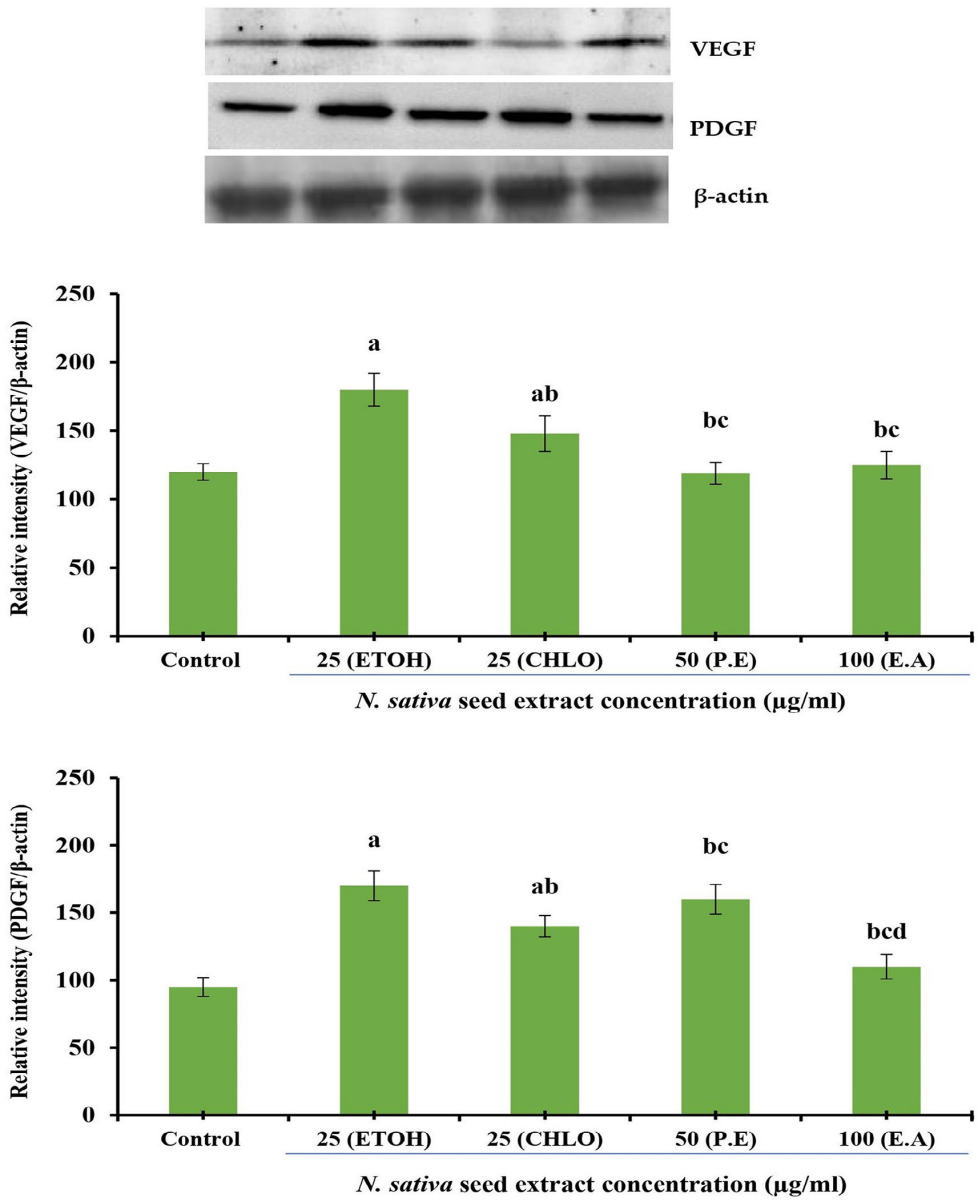
Effect of different *N. sativa* seed extracts on VEGF and PDGF protein expression in NHDF cells. Protein expression was analyzed by western blotting using specific antibodies. Each bar represents the mean ± SEM of five independent observations. Significance was considered as the levels of p < 0.05 level using Duncan’s multiple range test. a, compared with control; b, compared with ethanolic extract (25 μg) treated cells; c, compared with chloroform extract (25 μg) treated cells; *N. sativa*, *Nigella sativa* L.; VEGF, vascular endothelial growth factor; PDGF, platelet-derived growth factor; NHDF, normal human dermal fibroblast; P. E, Petroleum Ether extract; CHLO, Chloroform extract; E. A, Ethyl Acetate extract; ETOH, Ethanolic extract.

The effect of different solvent extracts of *N. sativa* seeds on VEGF and PDGF protein expression in NHDF cell lines were investigated. Incubation for 24 h with extracts of indicated concentrations was conducted in DMEM supplemented with 10% FBS using NHDF cells. Densitometry analysis was used to calculate protein expression, which is expressed in relative intensity. Internal control was performed using β-Actin. Based on five independent observations, each bar represents the mean and standard error of the mean. The significance level was determined by using Duncan’s multiple range test at p < 0.05. VEGF and PDGF expression levels were comparatively increased at 25 μg/mL by both ethanolic and chloroform extracts ( Figure 4).

*Effect of N. sativa seed extracts on VEGF and PDGF protein expression in HUVEC cells*

**Figure 5.**
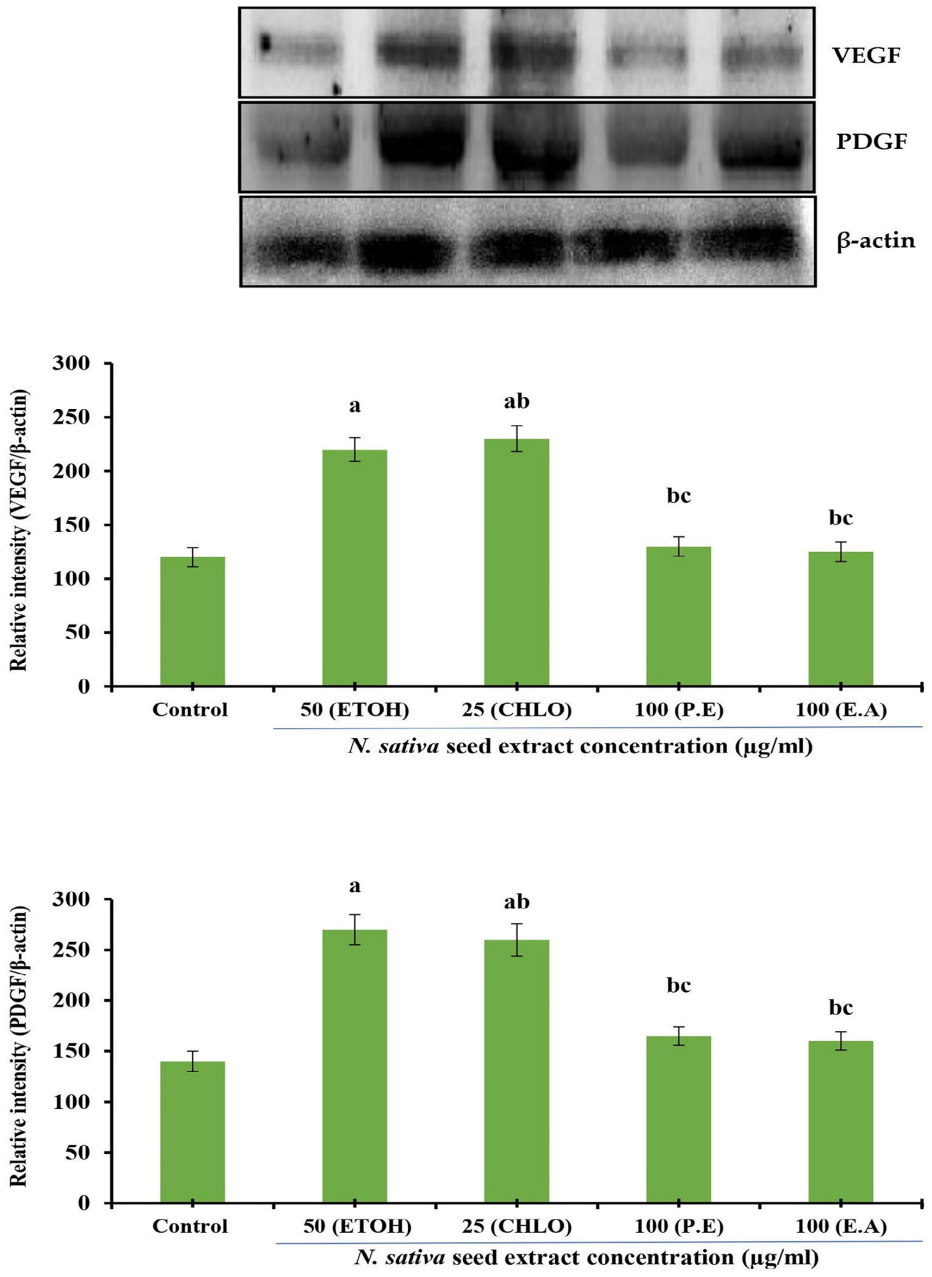
Effect of different *N. sativa* seed extracts on VEGF and PDGF protein expression in HUVEC cells. Protein expression was analyzed by western blotting using specific antibodies. Each bar represents the mean ± SEM of five independent observations. Significance was considered as the levels of p < 0.05 level using Duncan’s multiple range test. a, compared with control; b, compared with ethanolic extract (25 μg) treated cells; c, compared with chloroform extract (25 μg) treated cells; *N. sativa*, *Nigella sativa* L.; VEGF, vascular endothelial growth factor; PDGF, platelet-derived growth factor; HUVEC, human umbilical vein endothelial cell; P. E, Petroleum Ether extract; CHLO, Chloroform extract; E. A, Ethyl Acetate extract; ETOH, Ethanolic extract.

Effect of different solvent extracts on VEGF and PDGF protein expression in HUVEC lines. Incubation of HUVECs with indicated concentrations of extracts for 24 h was performed in DMEM supplemented with 10% FBS. Densitometry analysis was used to quantify protein expression, which is expressed as relative intensity. An internal control was performed using β-actin. Five independent observations are represented by a bar with a mean and standard error of measurement. The Duncan’s multiple range test was used to determine significance at p < 0.05. At a concentration of 25 μg/mL, ethanolic and chloroform extracts of *N. sativa* seed led to comparatively significant increases in the expression levels of VEGF and PDGF proteins ( Figure 5).

### *In silico* analysis

**Figure 6.**
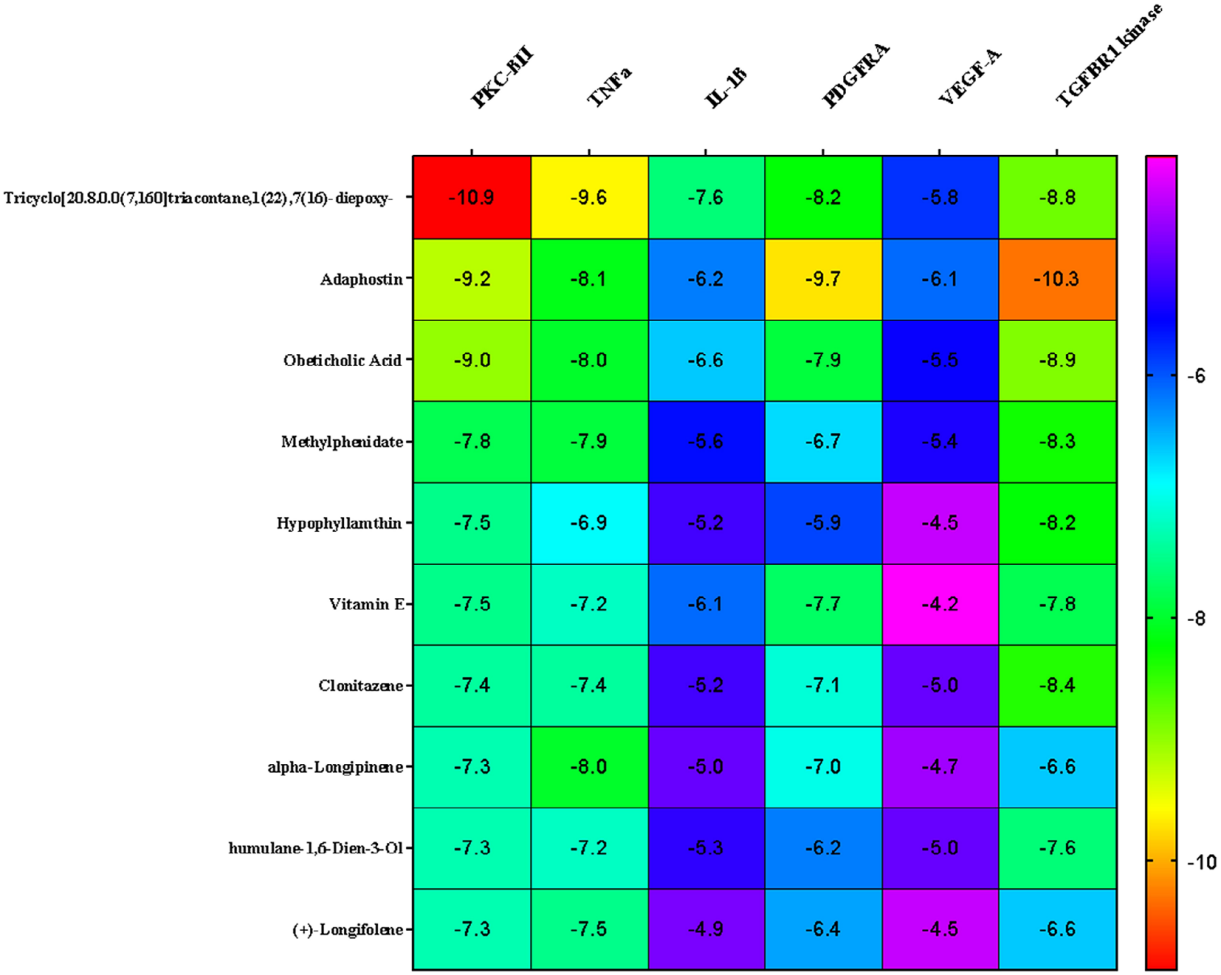
Heat map representation of top protein ligand docking analysis.

**Figure 7.**
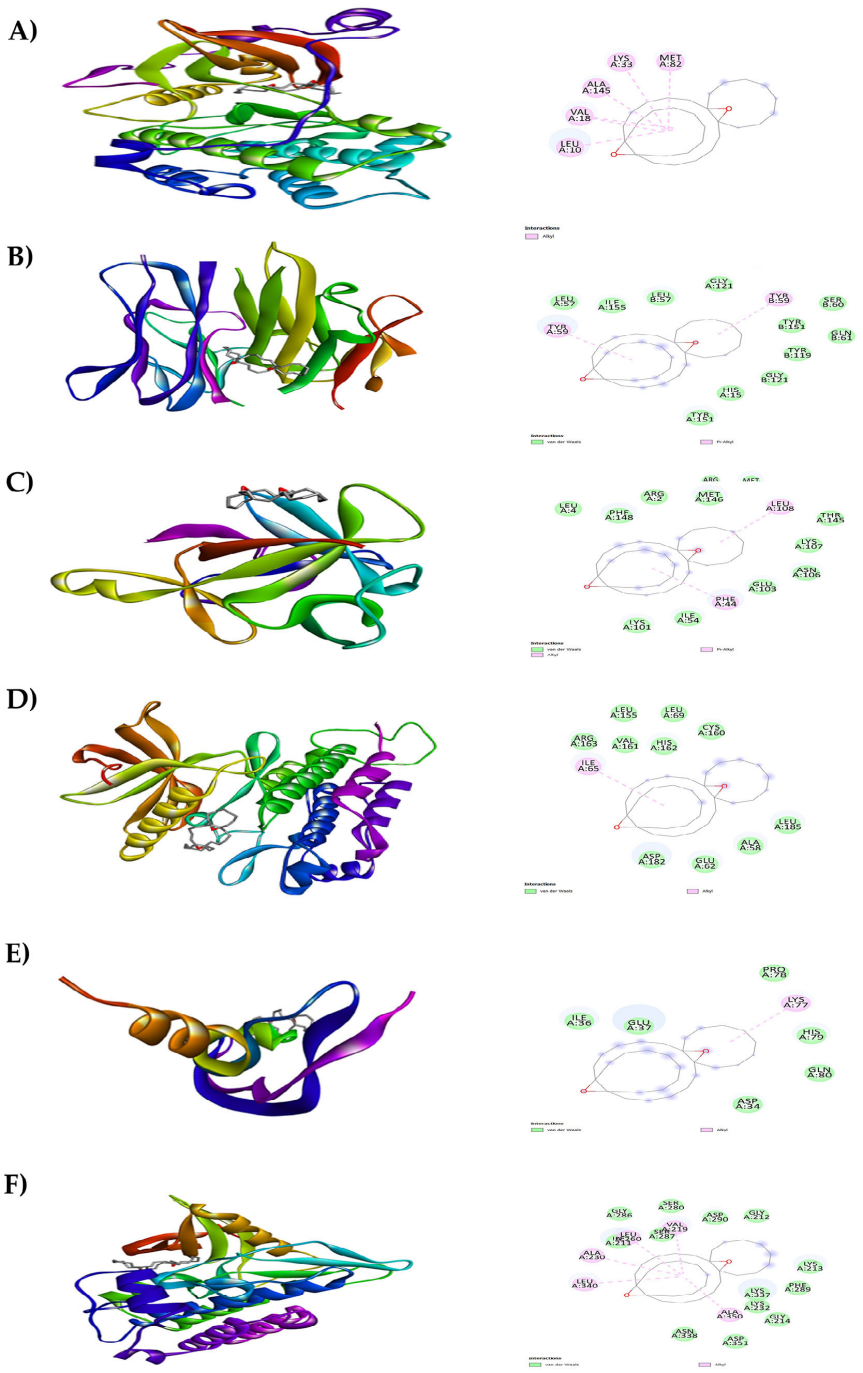
Results of molecular docking analysis. Molecular docking analysis of tricyclo[20.8.0.0(7,16)]triacontane, 1(22),7(16)-diepoxy- complexed with targeted proteins of A) PKC-βII, B) TNFα, C) IL-1β, D) PDGFRA, E) VEGF-A and F) TGFBR1.

**Figure 8.**
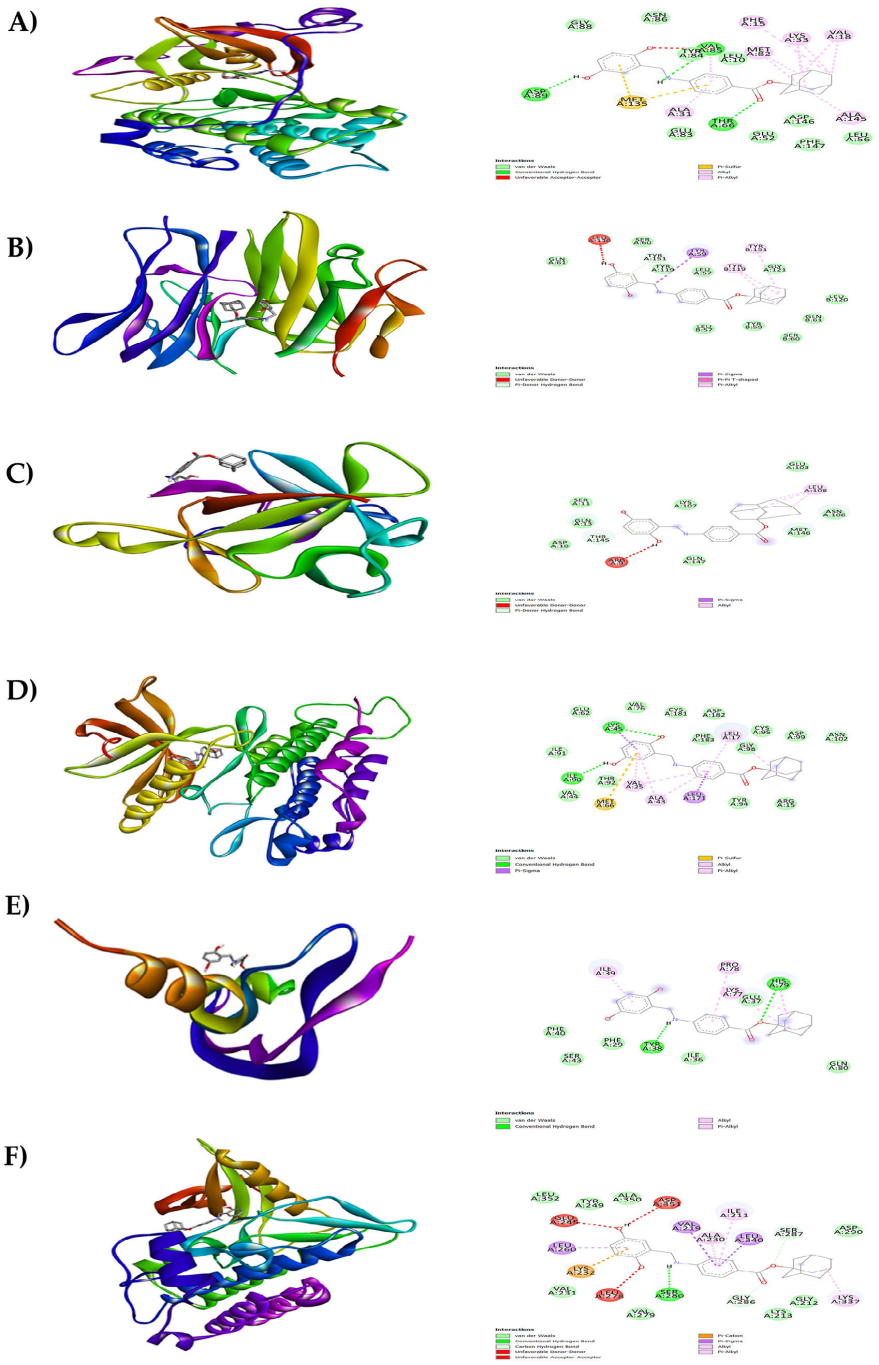
Results of molecular docking analysis. Molecular docking analysis of adaphostin complexed with targeted proteins of A) PKC-βII, B) TNFα, C) IL-1β, D) PDGFRA, E) VEGF-A and F) TGFBR1.

**Figure 9.**
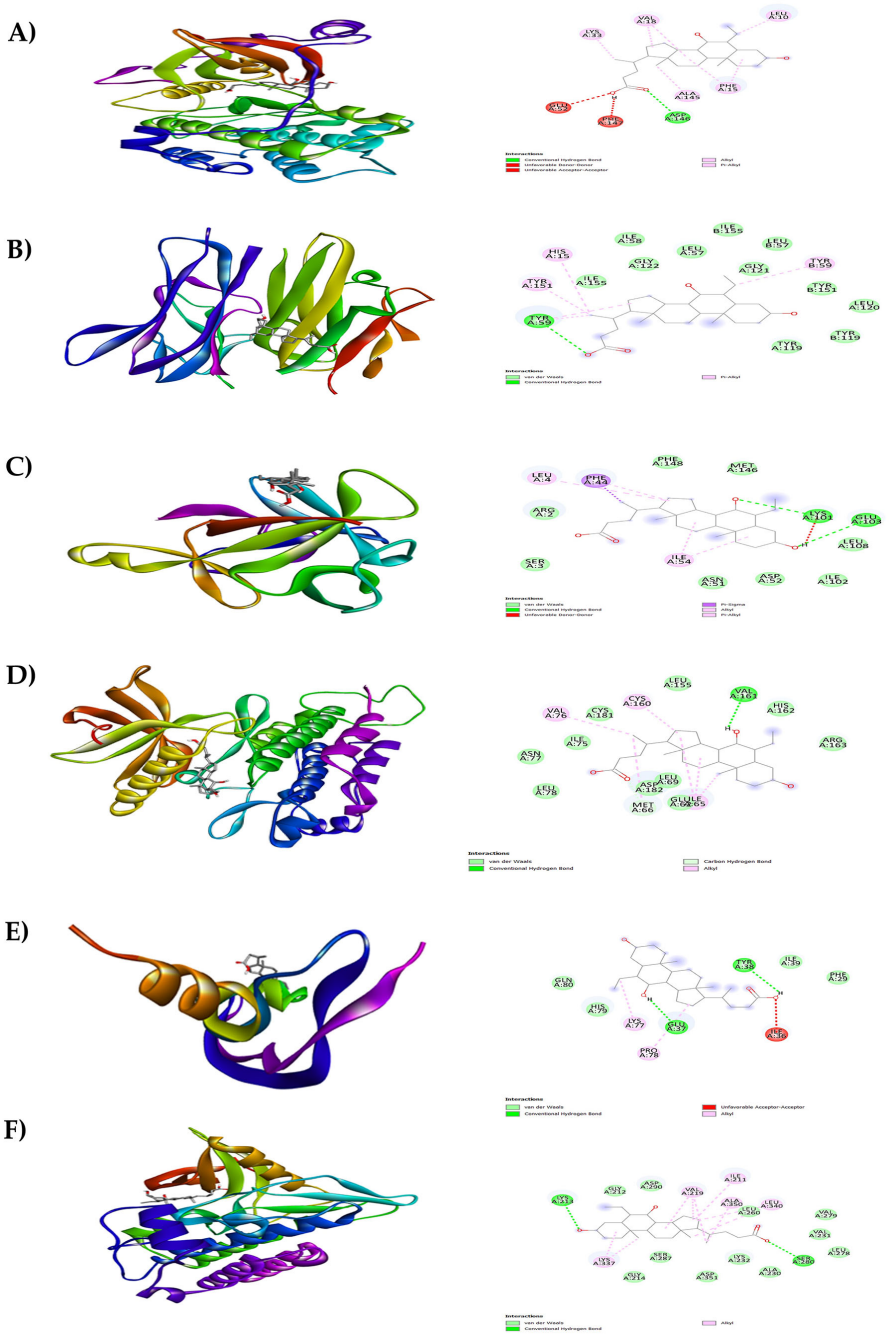
Results of molecular docking analysis. Molecular docking analysis of obeticholic acid complexed with targeted proteins of A) PKC-βII, B) TNFα, C) IL-1β, D) PDGFRA, E) VEGF-A and F) TGFBR1.

In the present study, Autodock Vina was used to predict the binding affinity of a total of 268 selected phytocompounds of *N. sativa* with the target proteins of wound healing process such as TNFα (PDB ID: 2AZ5), TGFBR1 kinase (PDB ID: 6B8Y), IL-1β (PDB ID: 6Y8M), PKC-βII (PDB: 2I0E), VEGF-A (PDB ID: 3QTK) and platelet-derived growth factor receptor alpha (PDGFRA) (PDB ID: 6JOL). The binding energies of 10 ligands that showed the highest binding affinities are indicated in the heatmap ( Figure 6). It is clear from Figure 6, three compounds (1, 2, and 3) namely tricyclo[20.8.0.0(7,16)]triacontane, 1(22),7(16)-diepoxy- (PubChem CID: 543764), adaphostin (PubChem CID: 387042), and obeticholic acid (PubChem CID: 447715), respectively, showed highest binding affinity with all the tested target proteins. The best docked protein ligand interactions are shown in Table 1 and Table 2. The 2D and 3D structures of the top docked complexes are shown in Figures 7- 9. ADME properties of those compounds were under the acceptable range ( Table 3).

**Table 1.** Molecular docking analysis of screened compounds with targeted proteins.

Compound name (PubChem CID)	AutoDock Score					
	PKC-βII (2I0E)	TNFα (2AZ5)	IL-1β (6Y8M)	PDGFRA (6JOL)	VEGF-A (3QTK)	TGFBR1 (6B8Y)
Tricyclo[20.8.0.0(7,16)]triacontane, 1(22),7(16)-diepoxy- (543764)	**-10.9**	**-9.6**	**-7.6**	**-8.2**	**-5.8**	**-8.8**
Adaphostin (387042)	**-9.2**	**-8.1**	**-6.2**	**-9.7**	**-6.1**	**-10.3**
Obeticholic acid (447715)	**-9.0**	**-8.0**	**-6.6**	**-7.9**	**-5.5**	**-8.9**
Methylphenidate, HFB (91747145)	-7.8	-7.9	-5.6	-6.7	-5.4	-8.3
Hypophyllanthin (182140)	-7.5	-6.9	-5.2	-5.9	-4.5	-8.2
Vitamin E (14985)	-7.5	-7.2	-6.1	-7.7	-4.2	-7.8
Clonitazene (62528)	-7.4	-7.4	-5.2	-7.1	-5.0	-8.4
α-Longipinene (520957)	-7.3	-8.0	-5.0	-7.0	-4.7	-6.6
Humulane-1,6-dien-3-ol (5353015)	-7.3	-7.2	-5.3	-6.2	-5.0	-7.6
(+)-Longifolene (1796220)	-7.3	-7.5	-4.9	-6.4	-4.5	-6.6

**Table 2.** Top protein-ligand docking complex interactions in the active sites.

Interaction residues	Distance	Category	Types
**PKC-βII complexed with tricyclo[20.8.0.0(7,16)]triacontane, 1(22),7(16)-diepoxy-**			
LEU10 VAL18 VAL18 LYS33 MET82 ALA145	5.01131 5.47023 4.98992 5.44287 4.97728 4.46926	Hydrophobic Hydrophobic Hydrophobic Hydrophobic Hydrophobic Hydrophobic	Alkyl Alkyl Alkyl Alkyl Alkyl Alkyl
**TNFα complexed with tricyclo[20.8.0.0(7,16)]triacontane, 1(22),7(16)-diepoxy-**			
TYR59 TYR59	4.46433 5.31031	Hydrophobic Hydrophobic	Pi-Alkyl Pi-Alkyl
**IL-1β complexed with tricyclo[20.8.0.0(7,16)]triacontane, 1(22),7(16)-diepoxy-**			
LEU108 PHE44	4.8333 4.68795	Hydrophobic Hydrophobic	Alkyl Pi-Alkyl
**PDGFRA complexed with tricyclo[20.8.0.0(7,16)]triacontane, 1(22),7(16)-diepoxy-**			
ILE65	5.13132	Hydrophobic	Alkyl
**VEGF-A complexed with tricyclo[20.8.0.0(7,16)]triacontane, 1(22),7(16)-diepoxy-**			
LYS77	4.91527	Hydrophobic	Alkyl
**TGFBR1 kinase complexed with tricyclo[20.8.0.0(7,16)]triacontane, 1(22),7(16)-diepoxy-**			
VAL219 ALA230 LEU260 LEU340 ALA350	4.46222 5.2525 5.30162 5.03535 4.70449	Hydrophobic Hydrophobic Hydrophobic Hydrophobic Hydrophobic	Alkyl Alkyl Alkyl Alkyl Alkyl
**PKC-βII complexed with adaphostin**			
THR66 VAL85 ASP89 MET135 MET135 VAL18 LYS33 LYS33 MET82 ALA145 MET82 VAL18 LYS33 PHE15 ALA31 VAL85	2.8462 2.83916 2.08593 4.31632 3.86336 4.67941 5.4335 4.02333 5.17432 4.50014 4.19605 4.38234 3.85054 5.32592 3.89466 5.13226	Hydrogen Bond Hydrogen Bond Hydrogen Bond Other Other Hydrophobic Hydrophobic Hydrophobic Hydrophobic Hydrophobic Hydrophobic Hydrophobic Hydrophobic Hydrophobic Hydrophobic Hydrophobic	Conventional Hydrogen Bond Conventional Hydrogen Bond Conventional Hydrogen Bond Pi-Sulfur Pi-Sulfur Alkyl Alkyl Alkyl Alkyl Alkyl Alkyl Alkyl Alkyl Pi-Alkyl Pi-Alkyl Pi-Alkyl
**TNFα complexed with adaphostin**			
TYR151 TYR59 TYR151 TYR119 TYR119 TYR151	4.02119 3.95293 5.8628 5.29859 4.93237 5.48208	Hydrogen Bond Hydrophobic Hydrophobic Hydrophobic Hydrophobic Hydrophobic	Pi-Donor Hydrogen Bond Pi-Sigma Pi-Pi T-shaped Pi-Alkyl Pi-Alkyl Pi-Alkyl
**IL-1β complexed with adaphostin**			
THR145 THR145 LEU108 LEU108 LEU108	3.43485 3.76017 5.31577 4.56027 3.89181	Hydrogen Bond Hydrophobic Hydrophobic Hydrophobic Hydrophobic	Pi-Donor Hydrogen Bond Pi-Sigma Alkyl Alkyl Alkyl
**PDGFRA complexed with adaphostin**			
LYS45 ILE90 LYS45 LEU171 MET66 LEU17 LEU17 VAL25 ALA43 VAL25 ALA43 LYS45	2.9452 2.07672 2.72555 2.83413 5.90633 5.20756 4.23893 5.17683 4.72332 4.60138 5.20594 4.49156	Hydrogen Bond Hydrogen Bond Hydrophobic Hydrophobic Other Hydrophobic Hydrophobic Hydrophobic Hydrophobic Hydrophobic Hydrophobic Hydrophobic	Conventional Hydrogen Bond Conventional Hydrogen Bond Pi-Sigma Pi-Sigma Pi-Sulfur Alkyl Pi-Alkyl Pi-Alkyl Pi-Alkyl Pi-Alkyl Pi-Alkyl Pi-Alkyl
**VEGF-A complexed with adaphostin**			
HIS79 TYR38 LYS77 LYS77 HIS79 PRO78 ILE39	3.30248 2.0803 5.09656 4.90891 4.92344 5.47674 3.81041	Hydrogen Bond Hydrogen Bond Hydrophobic Hydrophobic Hydrophobic Hydrophobic Hydrophobic	Conventional Hydrogen Bond Conventional Hydrogen Bond Alkyl Alkyl Pi-Alkyl Pi-Alkyl Pi-Alkyl
**TGFBR1 kinase complexed with adaphostin**			
SER280 GLY286 SER287 LYS232 VAL219 LEU260 LEU340 LYS337 ILE211 ALA230 LYS232	2.75731 3.52311 3.75326 4.0013 3.85337 3.84277 3.95459 4.03168 5.34457 5.22616 4.61046	Hydrogen Bond Hydrogen Bond Hydrogen Bond Electrostatic Hydrophobic Hydrophobic Hydrophobic Hydrophobic Hydrophobic Hydrophobic Hydrophobic	Conventional Hydrogen Bond Carbon Hydrogen Bond Carbon Hydrogen Bond Pi-Cation Pi-Sigma Pi-Sigma Pi-Sigma Alkyl Pi-Alkyl Pi-Alkyl Pi-Alkyl
**PKC-βII complexed with obeticholic acid**			
ASP146 VAL18 VAL18 ALA145 LYS33 LEU10 PHE15 PHE15	2.90673 5.21721 3.82528 3.10588 4.49913 4.55211 4.14543 4.92236	Hydrogen Bond Hydrophobic Hydrophobic Hydrophobic Hydrophobic Hydrophobic Hydrophobic Hydrophobic	Conventional Hydrogen Bond Alkyl Alkyl Alkyl Alkyl Alkyl Pi-Alkyl Pi-Alkyl
**TNFα complexed with obeticholic acid**			
TYR59 HIS15 TYR59 TYR59 TYR151 TYR59	3.01852 5.22368 3.82176 4.23758 4.27561 4.18769	Hydrogen Bond Hydrophobic Hydrophobic Hydrophobic Hydrophobic Hydrophobic	Conventional Hydrogen Bond Pi-Alkyl Pi-Alkyl Pi-Alkyl Pi-Alkyl Pi-Alkyl
**IL-1β with obeticholic acid**			
LYS101 GLU103 PHE44 ILE54 ILE54 LEU4 PHE44	2.78583 3.26326 3.61118 5.26877 5.16643 5.43239 4.93858	Hydrogen Bond Hydrogen Bond Hydrophobic Hydrophobic Hydrophobic Hydrophobic Hydrophobic	Conventional Hydrogen Bond Conventional Hydrogen Bond Pi-Sigma Alkyl Alkyl Alkyl Pi-Alkyl
**PDGFRA with obeticholic acid**			
VAL161 MET66 ILE65 CYS160 ILE65 ILE65 MET66 VAL76	2.55777 2.60333 4.98082 5.23492 5.20837 4.90546 4.8268 4.91524	Hydrogen Bond Hydrogen Bond Hydrophobic Hydrophobic Hydrophobic Hydrophobic Hydrophobic Hydrophobic	Conventional Hydrogen Bond Carbon Hydrogen Bond Alkyl Alkyl Alkyl Alkyl Alkyl Alkyl
**VEGF-A with obeticholic acid**			
A:TYR38 GLU37 PRO78 LYS77	2.2914 2.05672 5.08241 4.12618	Hydrogen Bond Hydrogen Bond Hydrophobic Hydrophobic	Conventional Hydrogen Bond Conventional Hydrogen Bond Alkyl Alkyl
**TGFBR1 kinase with obeticholic acid**			
LYS213 SER280 VAL219 VAL219 LYS337 ALA350 ILE211 LEU340 LEU340 LYS337 VAL219	3.18529 2.94006 5.27115 3.93297 5.0964 4.27292 5.2547 5.15809 4.34364 3.41833 5.19865	Hydrogen Bond Hydrogen Bond Hydrophobic Hydrophobic Hydrophobic Hydrophobic Hydrophobic Hydrophobic Hydrophobic Hydrophobic Hydrophobic	Conventional Hydrogen Bond Conventional Hydrogen Bond Alkyl Alkyl Alkyl Alkyl Alkyl Alkyl Alkyl Alkyl Alkyl

**Table 3.** ADME properties of screened phytocompounds.

Compound name (PubChem CID)	Mol. Wt	H. Bond Donor	H. Bond Acceptor	Log P	Rotatable Bond
Tricyclo[20.8.0.0(7,16)]triacontane, 1(22),7(16)-diepoxy- (543764)	444.7	0	2	9.5	0
Adaphostin (387042)	393.5	3	5	5.2	6
Obeticholic Acid (447715)	420.6	3	4	5.7	5

## Discussion

A wound is defined as a disruption of anatomical integrity of any biological tissue by physical, mechanical, chemical, or microbial factors. The wound healing process starts following the wound formation and repairs the injured or damaged tissues. ^28^ Development of natural wound healing agents is of current interest to mitigate the side effects of wound care products. ^29^ Nature has gifted us with a diverse range of medicinal plants to treat various ailments including wound healing. ^30^ It has been reported that *N. sativa* has a wide range of pharmaceutical properties. ^31^ The complete extracts and their phytocompounds from *N. sativa* seeds have not been investigated for their wound healing properties. Therefore, this study has been conducted to determine wound healing activity of various solvent extracts of *N. sativa* seeds by *in vitro* and *in silico* analyses. Cells, cellular components, and chemical mediators interact to heal wounds in a complex way. ^32^ The process of wound healing is broadly divided into four phases, namely coagulation and hemostasis, inflammation, proliferation, and scar tissue formation (maturation). The process of angiogenesis involves the formation of new blood vessels, and it is one of the most important steps in wound healing. ^33^ In the wound area, angiogenic signals from the macrophage-derived factors stimulate the proliferation, migration and differentiation of endothelial cells, and subsequent increase in blood vessel formation. ^34^ During the wound healing process, the new capillaries develop into the fibrin clots, which subsequently form a microvascular network that is a critical for the formation tissue formation. HUVECs are primary endothelial cells from umbilical cord and are widely used for *in vitro* investigation of angiogenesis. ^35^ In order to determine whether different crude extracts of *N. sativa* are cytotoxic, this study first carried out MTS tests on HUVECs. As shown in the Figure 2, all tested crude extracts did not exert any cytotoxic activity on the HUVECs. Notably, ethanol and chloroform extracts significantly enhanced the viability of HUVECs. Angiogenesis involves a complex series of molecular events mediated by several factors. In the wound healing process, there are a number of growth factors that play key roles, including PDGF, TGF-β1, EGF, VEGF and bFGF. ^36^ Proangiogenic factors, such as VEGF, promote the survival, migration, differentiation, self-assembly, and self-repair of endothelial cells. As soon as VEGF binds to the VEGF receptor, multiple downstream protein kinase pathways are activated and new blood vessels are formed. ^37^ The wound healing process is also affected by PDGF, another important growth factor. Additionally, PDGF stimulates the formation of new blood vessels by acting as a pro-angiogenic factor. ^38^ Thus, the onset of angiogenesis is positively regulated by both PDGF and VEGF. Therefore, this study has analyzed the expression levels of VEGF and PDGF in both tested cell lines. As seen in Figures 4 and 5, both ethanol and chloroform extracts increased the expression levels of VEGF and PDGF in both NHDFs as well as HUVECs. This indicates that the *N. sativa* seed extracts might promote the cell survival and self-repair of cells, and subsequent wound healing efficacy.

Wounds are characterized by excessive inflammation due to increased local and systemic levels of TNFα. ^39^ Evidence suggests that inhibition of TNFα is critical for the treatment of wounds. It plays an important role in wound healing by re-epithelializing, inducing inflammation, stimulating angiogenesis, and forming new skin tissue. ^40^ The docking studies were used to predict the possible therapeutic effects of phytocompounds of *N. sativa* against wound healing related molecular targets including TNFα, TGFBR1 kinase, IL-1β, PKC-βII, VEGF and PDGF. Based on the docking studies, it was predicted that bioactive compounds *N. sativa* showed strong binding affinity to select the wound healing related targets. Together, the current study results suggest that *N. sativa* seeds might exert wound healing effects mainly through the modulation of proangiogenic factors.

## Conclusions

Management of chronic wounds and the development of natural wound healing products is of critical importance in the area of clinical research. Medicinal plants have long served as a potential source of wound healing medications since ancient times, with their use going as far back as 3,000 BC). ^41^ *N. sativa* is one such medicinal herb that has been shown to possess a wide range of pharmacological properties. In this aspect, this study investigated the wound healing properties of different solvent extracts of *N. sativa* seeds. Both ethanolic and chloroform extracts significantly improved the viability in NHDF and HUVEC cell lines. Besides, both ethanolic and chloroform extracts increased the expression levels of VEGF and PDGF proteins indicating *N. sativa* can have significant impact on the rate of wound healing by promoting the angiogenesis and cell proliferation. The computational analysis of identified phytocompounds from the GC-MS spectrum showed potent binding affinity towards the wound healing-associated target proteins such as PKC-βII, TNFα, IL-1β, PDGFRA, VEGF-A, and TGFBR1 kinase. Based on the current findings, *N. sativa* seed extracts can exert potent wound healing activity *via* activating the VEGF and PDGF signaling pathways. However, further *in vitro* and *in vivo *studies are still required to confirm the current findings.

## Data Availability

Zenodo: *Nigella sativa* L. seed extracts promotes wound healing progress by activating VEGF and PDGF signalling pathways: An *in vitro* and *in silico* study, https://doi.org/10.5281/zenodo.7712528. ^42^ This project contains the following data:
• Cell viability assay.zip • Docking results.zip • GC-MS identified Compounds from *N. sativa* seed extracts.zip • Protein structures.zip • Westernblot raw data.pptx • List of tables.docx Data are available under the terms of the Creative Commons Attribution 4.0 International license (CC-BY 4.0).
