# Supplementary material for: Nigella sativa L. seed extracts promote wound healing progress by activating VEGF and PDGF signaling pathways: An in vitro and in silico study
Source: F1000Res. 2023 Jun 21;12:436. Originally published 2023 Apr 24. [Version 2] doi: 10.12688/f1000research.132106.2 (PMC10230177; doi:10.12688/f1000research.132106.2)
Supplement: Peer review history

# Peer review: Reviewer response for version 2

**DOI:** 10.5256/f1000research.151105.r180406

**Published:** 2023-07-06

**Authors:** Viji Rajendran

**Affiliations:** 1 State Key Laboratory of Water Environmental Simulation, School of Environment, Beijing Normal University, Beijing, China

As per the reviewer comments, author carefully revised and addressed the reviewer comments. I approved and recommend this research manuscript for the publication.

Is the work clearly and accurately presented and does it cite the current literature?

Yes

If applicable, is the statistical analysis and its interpretation appropriate?

Yes

Are all the source data underlying the results available to ensure full reproducibility?

Yes

Is the study design appropriate and is the work technically sound?

Yes

Are the conclusions drawn adequately supported by the results?

Yes

Are sufficient details of methods and analysis provided to allow replication by others?

Yes

Reviewer Expertise:

Environmental Science

I confirm that I have read this submission and believe that I have an appropriate level of expertise to confirm that it is of an acceptable scientific standard.

# Peer review: Reviewer response for version 2

**DOI:** 10.5256/f1000research.151105.r180407

**Published:** 2023-06-30

**Authors:** Monica Mironescu

**Affiliations:** 1 Faculty of Agricultural Sciences Food Industry and Environmental Protection, Lucian Blaga University of Sibiu, Sibiu, Romania

The authors revised their manuscript as required.

Is the work clearly and accurately presented and does it cite the current literature?

Yes

If applicable, is the statistical analysis and its interpretation appropriate?

Yes

Are all the source data underlying the results available to ensure full reproducibility?

Yes

Is the study design appropriate and is the work technically sound?

Yes

Are the conclusions drawn adequately supported by the results?

Yes

Are sufficient details of methods and analysis provided to allow replication by others?

Yes

Reviewer Expertise:

microbiology, antimicrobials

I confirm that I have read this submission and believe that I have an appropriate level of expertise to confirm that it is of an acceptable scientific standard.

# Peer review: Reviewer response for version 1

**DOI:** 10.5256/f1000research.145007.r171687

**Published:** 2023-06-20

**Authors:** Vinothkannan Ravichandran

**Affiliations:** 1 Amity University, Mumbai, Panvel, Maharashtra, India

Here the authors found that *Nigella sativa* L. seed extracts promote wound healing progress by activating VEGF and PDGF signaling pathways.

- *N. sativa* seeds were purchased from a local herbal shop in Bangkok, Thailand. But was it authenticated? If yes, that detail has to be included. 
- HUVEC and NHDF cell lines were used, explain why they have been chosen.
- Yield of the extraction was not mentioned anywhere; it has to be mentioned.
- Chromatogram image is not clear. Update the image.
- Effect of extracts on HUVEC viability figure is a little confusing because of the comparison. Explain what extracts in legend (NSE something like that).
- Docking data Table 1 has to include chemical structure of the compound. 
- Discussion has to be elaborated on.

Is the work clearly and accurately presented and does it cite the current literature?

Yes

If applicable, is the statistical analysis and its interpretation appropriate?

Yes

Are all the source data underlying the results available to ensure full reproducibility?

Yes

Is the study design appropriate and is the work technically sound?

Yes

Are the conclusions drawn adequately supported by the results?

Yes

Are sufficient details of methods and analysis provided to allow replication by others?

Yes

Reviewer Expertise:

Chromatography, Cell line studies, Docking

I confirm that I have read this submission and believe that I have an appropriate level of expertise to confirm that it is of an acceptable scientific standard, however I have significant reservations, as outlined above.

# Peer review: Reviewer response for version 1

**DOI:** 10.5256/f1000research.145007.r171688

**Published:** 2023-05-30

**Authors:** Viji Rajendran

**Affiliations:** 1 State Key Laboratory of Water Environmental Simulation, School of Environment, Beijing Normal University, Beijing, China

In this study, the authors examined the wound healing properties of *Nigella sativa* seed extracts using normal cell lines such as normal human dermal fibroblasts (NHDFs) and normal human umbilical vein endothelia cells (HUVECs). An analysis of western blots was performed to determine VEGF and PDGF expression. Moreover, computational analysis was conducted to screen the potential bioactive compounds for wound healing. The overall quality of the research work and its clarity are excellent. The figures are clear and of good quality; in fact, they are intended to improve the reader's experience with the paper. The work is sufficiently scientifically interesting and technically sound to merit indexing.

 Although there are many minor corrections that need to be made, the authors should take time to revise the manuscript carefully.

1. Provide a detailed description of the incubation period, treatment period of the cells, and concentration of the extracts used in the cell viability analysis.
2. The international unit system should be checked and corrected throughout the manuscript.
3. The author stated that each bar represented the mean + SEM of six independent observations, but the figure only shows five independent bars. The author should correct the figures.
4. There is a need for the author to clarify whether the treatment period was 24 or 48 hours.
5. ‘Effect of crude seed extracts on VEGF and PDGF protein expression in HUVEC lines’ it should be ‘Effect of crude seed extracts on VEGF and PDGF protein expression in HUVEC cell line’.

Is the work clearly and accurately presented and does it cite the current literature?

Yes

If applicable, is the statistical analysis and its interpretation appropriate?

Yes

Are all the source data underlying the results available to ensure full reproducibility?

Yes

Is the study design appropriate and is the work technically sound?

Yes

Are the conclusions drawn adequately supported by the results?

Yes

Are sufficient details of methods and analysis provided to allow replication by others?

Yes

Reviewer Expertise:

Environmental Science

I confirm that I have read this submission and believe that I have an appropriate level of expertise to confirm that it is of an acceptable scientific standard.

# Peer review: Reviewer response for version 1

**DOI:** 10.5256/f1000research.145007.r171685

**Published:** 2023-05-18

**Authors:** Monica Mironescu

**Affiliations:** 1 Faculty of Agricultural Sciences Food Industry and Environmental Protection, Lucian Blaga University of Sibiu, Sibiu, Romania

*Nigella sativa* is native to Southeast Asia and its seed extract has been used for many centuries to treat diseases and more recently, the main active compound - thymoquinone has been tested for its effectiveness against diseases such as cancer. There are numerous researches and reviews that evaluate the medicinal use of *Nigella sativa* in various conditions. A series of preclinical and clinical studies describe multiple effects of *Nigella sativa* in animal or cellular models such as bronchodilator, antihistaminic, anti-inflammatory, anti-leukotriene and immunomodulatory effects.

 This research is investigating the action of *Nigella sativa* seed extracts on wound healing and its progress by activating VEGF and PDGF signaling pathways. In vitro and in silico studies are realized. The research is very complex and well done.

 Still, there are two aspects to be improved by the authors:

 1. There is not enough discussion of the results obtained by the authors in comparison with other researches.

 2. Some spelling mistakes are given below:

- To connect the time of the verb with the subject in the proposition: Infection by pathogenic bacteria delay the healing process and increase health risks to the general public;
- 'Aliments' instead of 'ailments';
- 'Tap water' instead of 'tab water';
- To finish the proposition: A humidified atmosphere containing 5% CO 2 was used for HUVEC and NHDF cell lines during the experiment to maintain 37°C for the duration …;
- Comma in the phrase "…as the control)" has to be removed.

Is the work clearly and accurately presented and does it cite the current literature?

Yes

If applicable, is the statistical analysis and its interpretation appropriate?

Yes

Are all the source data underlying the results available to ensure full reproducibility?

Yes

Is the study design appropriate and is the work technically sound?

Yes

Are the conclusions drawn adequately supported by the results?

Yes

Are sufficient details of methods and analysis provided to allow replication by others?

Yes

Reviewer Expertise:

microbiology, antimicrobials

I confirm that I have read this submission and believe that I have an appropriate level of expertise to confirm that it is of an acceptable scientific standard, however I have significant reservations, as outlined above.

# Peer review: Reviewer response for version 1

**DOI:** 10.5256/f1000research.145007.r171686

**Published:** 2023-05-16

**Authors:** Balasubramani Ravindran

**Affiliations:** 1 Department of Environmental Energy & Engineering, Kyonggi University, Suwon, South Korea

Palanisamy *et al.*, have demonstrated the role of Nigella sativa L. (N. sativa) seed extracts for wound healing potential using two different cell lines and with computational work in order to show the mechanisms via regulates VEGF and PDGF signalling. This research work has scientific merit and sounds enough to be considered in the F1000Research journal.

 However, the authors should justify the following:

1. Whether the authors have done phytochemical screening analysis in all the extracts?
2. What was the dilution of primary antibody? Please mention.
3. The plant name should be in italic style, please check it throughout the manuscript.
4. The author may continue these findings in *in vivo* experimental animal model.
5. How can the authors conclude N. sativa seed extracts possess wound healing properties with basic experiments? Please justify.

Is the work clearly and accurately presented and does it cite the current literature?

Yes

If applicable, is the statistical analysis and its interpretation appropriate?

Yes

Are all the source data underlying the results available to ensure full reproducibility?

Yes

Is the study design appropriate and is the work technically sound?

Yes

Are the conclusions drawn adequately supported by the results?

Yes

Are sufficient details of methods and analysis provided to allow replication by others?

Yes

Reviewer Expertise:

Plants

I confirm that I have read this submission and believe that I have an appropriate level of expertise to confirm that it is of an acceptable scientific standard.
